# Supplementary material for: A phenotypic screening platform utilising human spermatozoa identifies compounds with contraceptive activity
Source: eLife. 2020 Jan 28;9:e51739. doi: 10.7554/eLife.51739 (PMC7046468; doi:10.7554/eLife.51739)
Supplement: Figure 2—source data 1. — Compound names and nominal target/functional annotation shown on left along with chemical structure and physico-chemical properties. The 8-point dose response curves for each hit with estimated Hill slope, EC50 and Efficacy [% max reduction] are shown on the right. Two data points per concentration (n = 2); data points are Mean ± SD. A 4-parameter logistic fit has been performed using R package: dr4pl. Calculated EC50 values and Efficacy (% max effect) in the motility assay are shown in the right hand section. CC50 values for HEK293 and HepG2 CellTiter-Glo cytotoxicity assays performed by CALIBR (www.reframedb.org) are shown in the right hand section. Note: 0 = inactive in cytotoxicity assay. The chemical structure of the hit compound is shown as well as some annotation and physicochemical properties (for nomenclature description see Figure 2). See Figure 2—source data 2 along with Figure 2—source code 2. [file elife-51739-fig2-data1.pdf]

Figure 2-figure supplement 1

Kf-4939
Platelet aggregation inhibitor

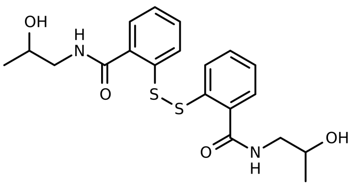

MW (RDKit)
420.118

QED (RDKit)
0.466

SlogP (RDKit)
2.707

TPSA (RDKit)
98.66

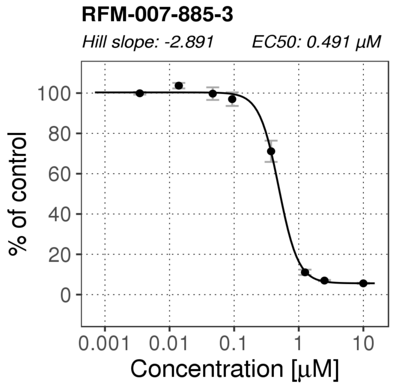

EC50 [μM]
0.49

Efficacy [% reduction]
100

Hek CC50 [μM]
2.526

HepG2 CC50 [μM]
0

Mercufenol Chloride
Antiseptic

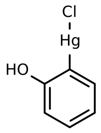

MW (RDKit)
329.974

QED (RDKit)
0.769

SlogP (RDKit)
1.254

TPSA (RDKit)
20.23

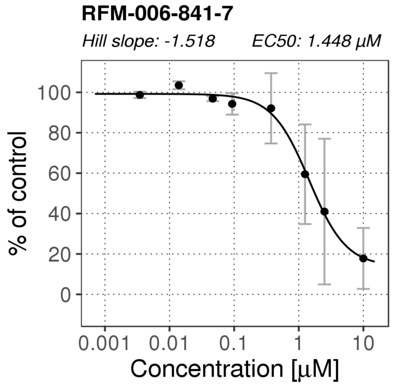

EC50 [μM]
1.45

Efficacy [% reduction]
98

Hek CC50 [μM]
0.595

HepG2 CC50 [μM]
2.721

Bismuth Ethanedithiol
Unidentified pharmacological action

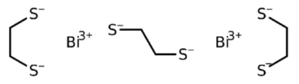

MW (RDKit)
392.932

QED (RDKit)
0.58

SlogP (RDKit)
2.382

TPSA (RDKit)
0

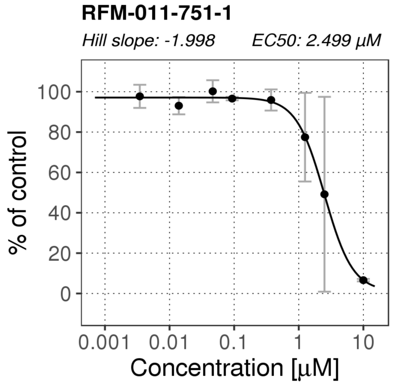

EC50 [μM]
2.5

Efficacy [% reduction]
98

Hek CC50 [μM]
2.716

HepG2 CC50 [μM]
1.141

Tyrothricin
Antibiotic

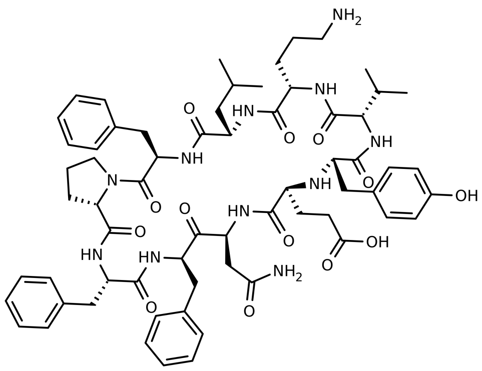

MW (RDKit)
1227.633

QED (RDKit)
0.063

SlogP (RDKit)
1.138

TPSA (RDKit)
379.75

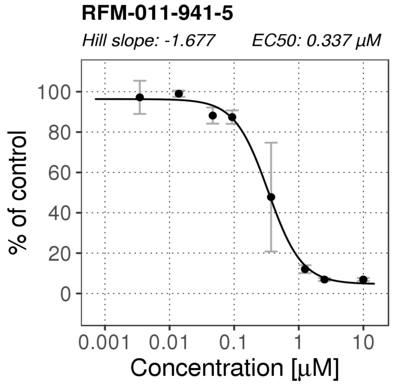

EC50 [μM]
0.34

Efficacy [% reduction]
97

Hek CC50 [μM]
1.791

HepG2 CC50 [μM]
0

Ticlatone
Antibiotic

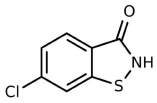

MW (RDKit)
184.97

QED (RDKit)
0.671

SlogP (RDKit)
2.243

TPSA (RDKit)
32.86

RFM-000-486-4
Hill slope: -1.247 *EC50: 4.435 µM*

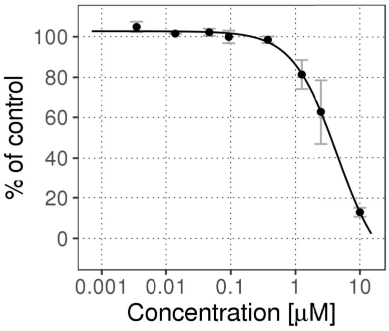

EC50 [µM]
4.43

Efficacy
[% reduction]
96

Hek CC50 [µM]
2.259

HepG2 CC50 [µM]
0

Phenylmercuric Borate
Antiseptic

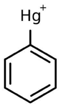

MW (RDKit)
279.009

QED (RDKit)
0.617

SlogP (RDKit)
0.859

TPSA (RDKit)
0

RFM-003-300-1
Hill slope: -2.357 *EC50: 1.589 µM*

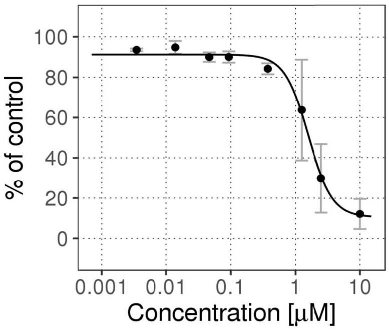

EC50 [µM]
1.59

Efficacy
[% reduction]
91

Hek CC50 [µM]
0.687

HepG2 CC50 [µM]
0

Alexidine
Phospholipase inhibitor

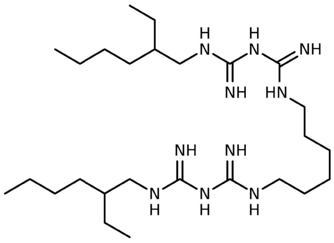

MW (RDKit)
508.469

QED (RDKit)
0.071

SlogP (RDKit)
4.256

TPSA (RDKit)
167.58

RFM-007-403-3
Hill slope: -1.695 *EC50: >10 µM*

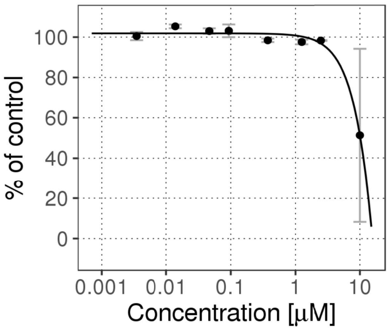

EC50 [µM]
> 10

Efficacy
[% reduction]
86

Hek CC50 [µM]
1.453

HepG2 CC50 [µM]
1.506

Disulfiram
Aldehyde dehydrogenase 2 inhibitor

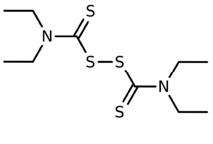

MW (RDKit)
296.051

QED (RDKit)
0.573

SlogP (RDKit)
3.621

TPSA (RDKit)
6.48

RFM-000-224-4
Hill slope: -1.755 *EC50: >10 µM*

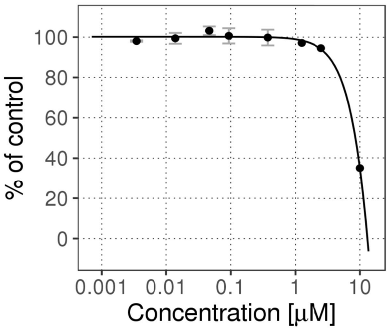

EC50 [µM]
> 10

Efficacy
[% reduction]
70

Hek CC50 [µM]
7.159

HepG2 CC50 [µM]
0

Octenidine
Antibacterial

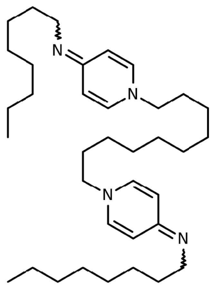

MW (RDKit)
550.497

QED (RDKit)
0.11

SlogP (RDKit)
9.633

TPSA (RDKit)
34.58

RFM-004-129-2
Hill slope: -1.615 *EC50: 6.845 µM*

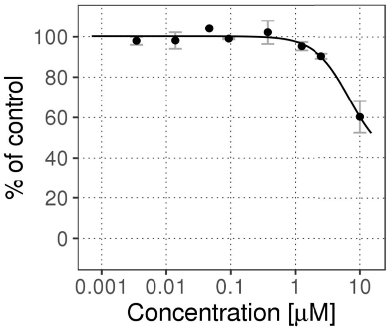

EC50 [µM]
6.85

Efficacy [% reduction]
54

Hek CC50 [µM]
0.798

HepG2 CC50 [µM]
1.763

Pfk-015
PFKFB3 inhibitor

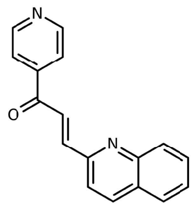

MW (RDKit)
260.095

QED (RDKit)
0.534

SlogP (RDKit)
3.526

TPSA (RDKit)
42.85

RFM-004-705-2
Hill slope: -0.625 *EC50: >10 µM*

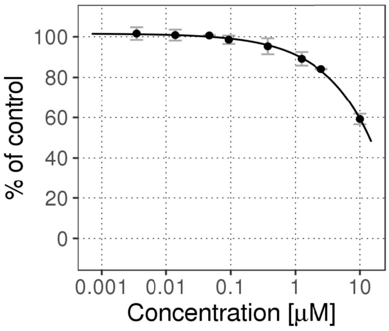

EC50 [µM]
> 10

Efficacy [% reduction]
47

Hek CC50 [µM]
1.382

HepG2 CC50 [µM]
0.95

Methylprednisolone Aceponate
Antiinflammatory

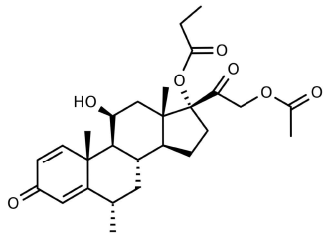

MW (RDKit)
472.246

QED (RDKit)
0.612

SlogP (RDKit)
3.335

TPSA (RDKit)
106.97

RFM-007-141-0
Hill slope: -1.126 *EC50: 2.521 µM*

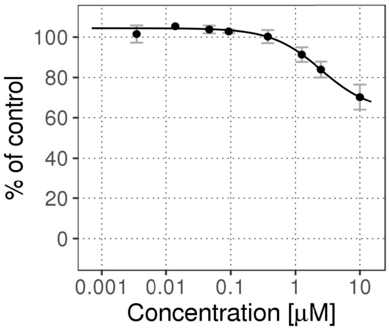

EC50 [µM]
2.52

Efficacy [% reduction]
45

Hek CC50 [µM]
0

HepG2 CC50 [µM]
0

Zoticasone Propionate
Glucocorticoid receptor agonist

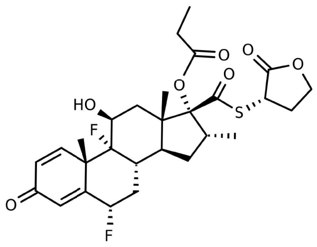

MW (RDKit)
552.199

QED (RDKit)
0.525

SlogP (RDKit)
3.819

TPSA (RDKit)
106.97

RFM-009-109-8
Hill slope: -1.199 *EC50: 2.385 µM*

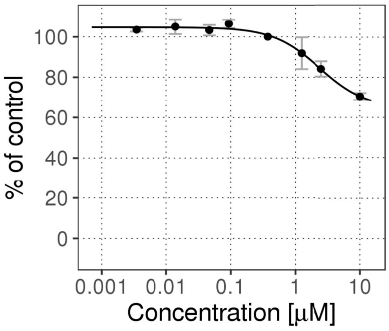

EC50 [µM]
2.38

Efficacy [% reduction]
39

Hek CC50 [µM]
0

HepG2 CC50 [µM]
0

Brigatinib
ALK receptor tyrosine kinase inhibitor

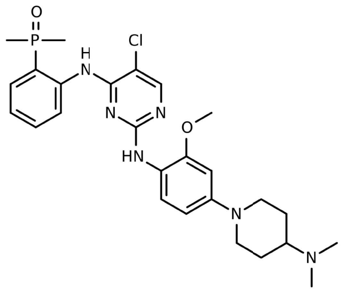

MW (RDKit)
528.217

QED (RDKit)
0.376

SlogP (RDKit)
5.404

TPSA (RDKit)
82.62

RFM-007-125-0
Hill slope: -2.921 *EC50: 0.680 µM*

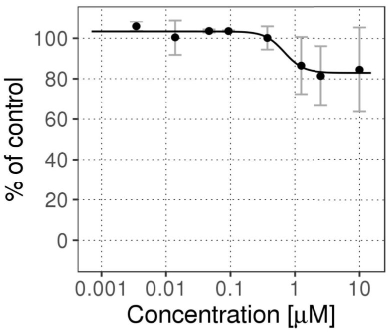

EC50 [µM]
0.68

Efficacy
[% reduction]
38

Hek CC50 [µM]
1.867

HepG2 CC50 [µM]
0.136

Fluorometholone Acetate
Antiinflammatory

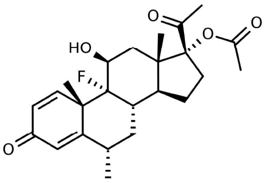

MW (RDKit)
418.216

QED (RDKit)
0.695

SlogP (RDKit)
3.494

TPSA (RDKit)
80.67

RFM-000-926-7
Hill slope: -1.038 *EC50: 3.304 µM*

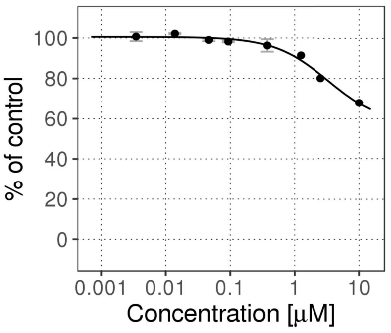

EC50 [µM]
3.3

Efficacy
[% reduction]
35

Hek CC50 [µM]
0

HepG2 CC50 [µM]
0

Amibegron Hydrochloride
beta3-Adrenoceptor Agonist

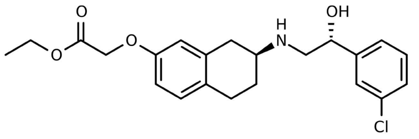

MW (RDKit)
403.155

QED (RDKit)
0.66

SlogP (RDKit)
3.462

TPSA (RDKit)
67.79

RFM-005-517-4
Hill slope: -1.170 *EC50: 0.218 µM*

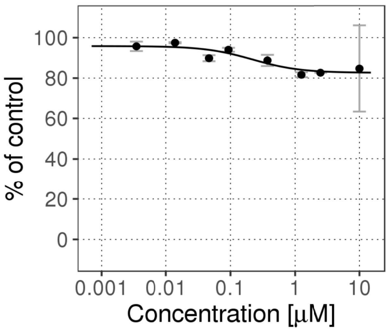

EC50 [µM]
0.22

Efficacy
[% reduction]
31

Hek CC50 [µM]
0

HepG2 CC50 [µM]
0

Uprosertib
PKB alpha/Akt1 inhibitor

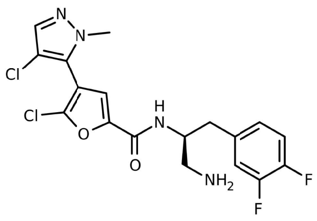

MW (RDKit)
428.062

QED (RDKit)
0.628

SlogP (RDKit)
3.565

TPSA (RDKit)
86.08

RFM-004-668-4
Hill slope: -0.595 *EC50: >10 µM*

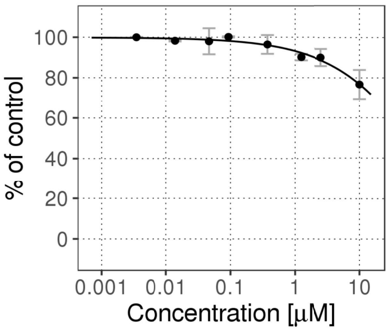

EC50 [µM]
> 10

Efficacy
[% reduction]
31

Hek CC50 [µM]
0

HepG2 CC50 [µM]
0

Kf-41399
Chemoprotective agent

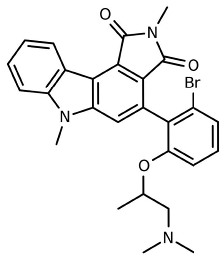

MW (RDKit)
519.116

QED (RDKit)
0.335

SlogP (RDKit)
5.316

TPSA (RDKit)
54.78

RFM-007-307-4
Hill slope: -1.221 *EC50: 4.428 µM*

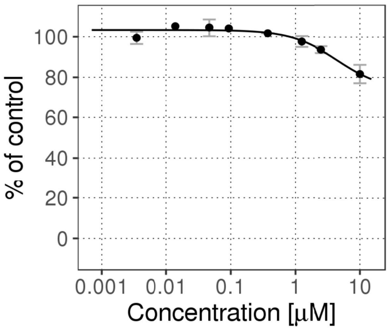

EC50 [µM]
4.43

Efficacy
[% reduction]
31

Hek CC50 [µM]
0

HepG2 CC50 [µM]
0

Halobetasol Propionate
Antisporiatic

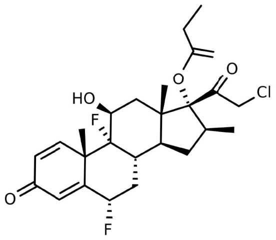

MW (RDKit)
484.183

QED (RDKit)
0.481

SlogP (RDKit)
4.051

TPSA (RDKit)
80.67

RFM-007-131-8
Hill slope: -0.576 *EC50: >10 µM*

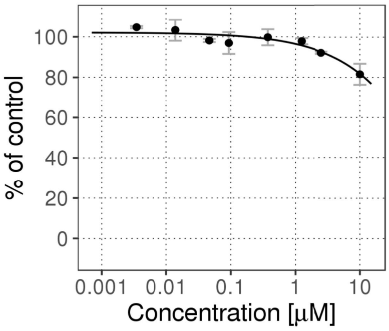

EC50 [µM]
> 10

Efficacy
[% reduction]
30

Hek CC50 [µM]
0

HepG2 CC50 [µM]
0

Pirtenidine
Antibiotic

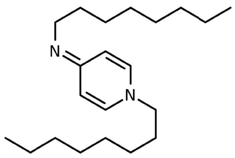

MW (RDKit)
318.303

QED (RDKit)
0.368

SlogP (RDKit)
6.11

TPSA (RDKit)
17.29

RFM-010-155-3
Hill slope: -0.825 *EC50: 2.662 µM*

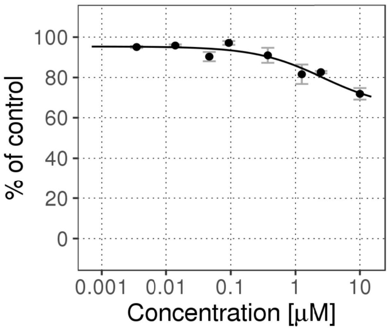

EC50 [µM]
2.66

Efficacy
[% reduction]
28

Hek CC50 [µM]
1.476

HepG2 CC50 [µM]
0

Lx-7101
LIM Domain Kinase inhibitor

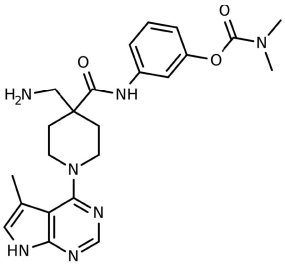

MW (RDKit)
451.233

QED (RDKit)
0.543

SlogP (RDKit)
2.511

TPSA (RDKit)
129.47

RFM-007-451-1
Hill slope: -0.760 *EC50: >10 µM*

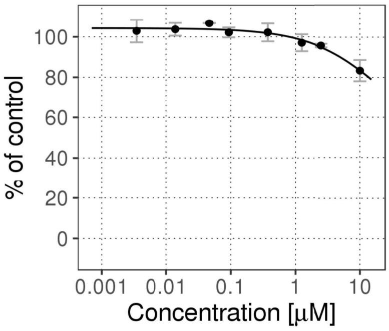

EC50 [µM]
> 10

Efficacy
[% reduction]
28

Hek CC50 [µM]
0

HepG2 CC50 [µM]
0

Ap26113-analog
ALK receptor tyrosine kinase inhibitor

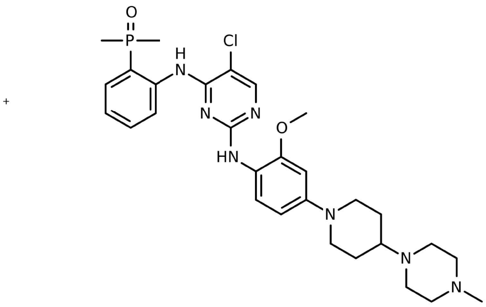

MW (RDKit)
528.217

QED (RDKit)
0.376

SlogP (RDKit)
5.404

TPSA (RDKit)
82.62

RFM-003-055-7
Hill slope: -0.695 *EC50: 1.353 µM*

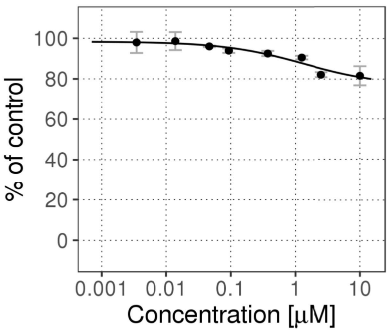

EC50 [µM]
1.35

Efficacy [% reduction]
24

Hek CC50 [µM]
0

HepG2 CC50 [µM]
0.13

Methylbenzethonium Chloride
Antiseptic

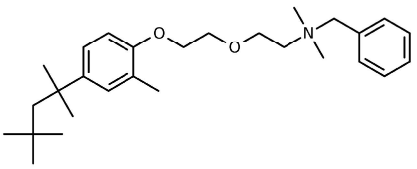

MW (RDKit)
426.337

QED (RDKit)
0.306

SlogP (RDKit)
6.381

TPSA (RDKit)
18.46

RFM-009-180-5
Hill slope: -1.134 *EC50: >10 µM*

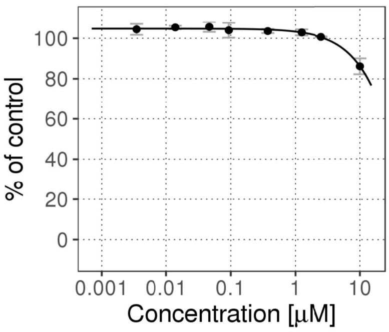

EC50 [µM]
> 10

Efficacy [% reduction]
24

Hek CC50 [µM]
3.65

HepG2 CC50 [µM]
7.586

Cabazitaxel
Tubulin inhibitor

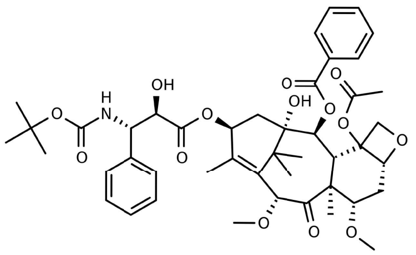

MW (RDKit)
835.378

QED (RDKit)
0.171

SlogP (RDKit)
4.568

TPSA (RDKit)
202.45

RFM-003-386-3
Hill slope: -0.144 *EC50: >10 µM*

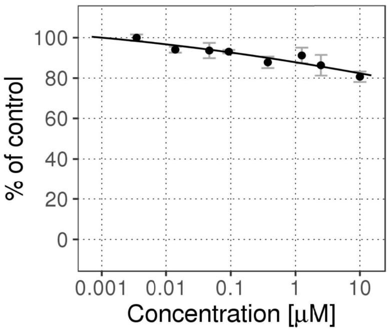

EC50 [µM]
> 10

Efficacy [% reduction]
23

Hek CC50 [µM]
0

HepG2 CC50 [µM]
0

Resiquimod
Immunostimulant

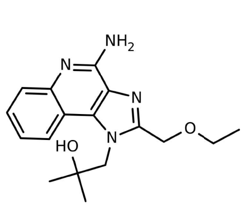

MW (RDKit)
314.174

QED (RDKit)
0.755

SlogP (RDKit)
2.474

TPSA (RDKit)
86.19

RFM-004-547-6
Hill slope: -4.142 *EC50: 3.217 µM*

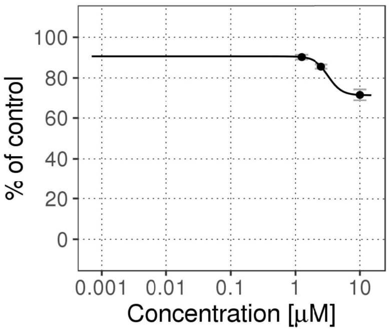

EC50 [µM]
3.22

Efficacy [% reduction]
22

Hek CC50 [µM]
0

HepG2 CC50 [µM]
0

Skq1
Neurologic drug

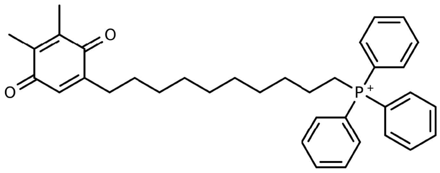

MW (RDKit)
537.292

QED (RDKit)
0.119

SlogP (RDKit)
7.906

TPSA (RDKit)
34.14

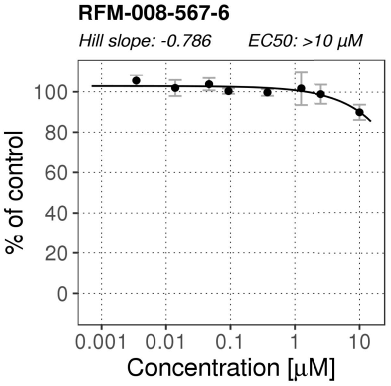

EC50 [µM]
> 10

Efficacy [% reduction]
21

Hek CC50 [µM]
0.778

HepG2 CC50 [µM]
4.41

Oxyphenbutazone
Treatment of Gout

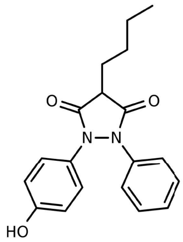

MW (RDKit)
324.147

QED (RDKit)
0.856

SlogP (RDKit)
3.493

TPSA (RDKit)
60.85

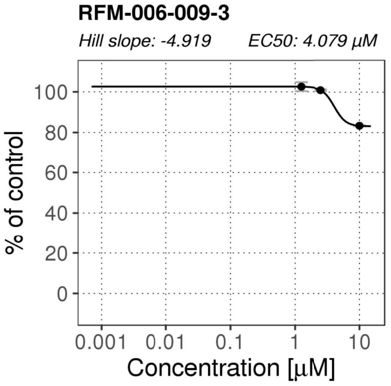

EC50 [µM]
4.08

Efficacy [% reduction]
21

Hek CC50 [µM]
1.572

HepG2 CC50 [µM]
0

Docetaxel
Tubulin inhibitor

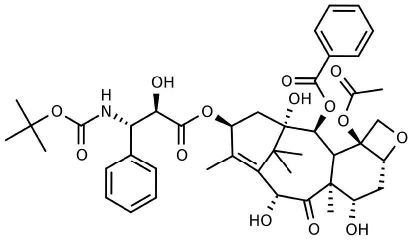

MW (RDKit)
807.347

QED (RDKit)
0.147

SlogP (RDKit)
3.26

TPSA (RDKit)
224.45

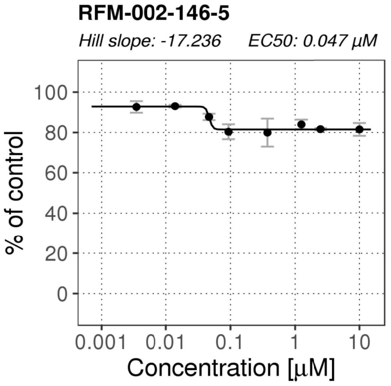

EC50 [µM]
0.05

Efficacy [% reduction]
20

Hek CC50 [µM]
0

HepG2 CC50 [µM]
0

Lestaurtinib
Flt3 inhibitor

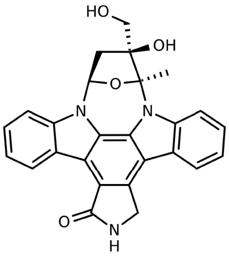

MW (RDKit)
439.153

QED (RDKit)
0.373

SlogP (RDKit)
3.474

TPSA (RDKit)
88.65

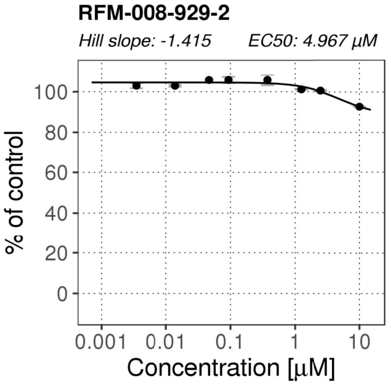

EC50 [µM]
4.97

Efficacy [% reduction]
17

Hek CC50 [µM]
0.551

HepG2 CC50 [µM]
0.029

Brigatinib

ALK receptor tyrosine kinase inhibitor

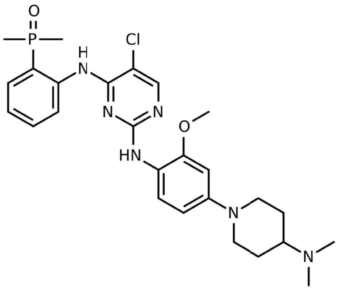

MW (RDKit)
583.259

QED (RDKit)
0.352

SlogP (RDKit)
5.09

TPSA (RDKit)
85.86

RFM-009-231-9

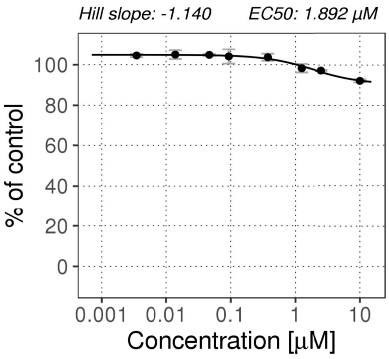

EC50 [µM]
1.89

Efficacy [% reduction]
15

Hek CC50 [µM]
8.066

HepG2 CC50 [µM]
0.435
