# Supplementary material for: A phenotypic screening platform utilising human spermatozoa identifies compounds with contraceptive activity
Source: eLife. 2020 Jan 28;9:e51739. doi: 10.7554/eLife.51739 (PMC7046468; doi:10.7554/eLife.51739)
Supplement: Figure 3—source data 2. — Compound names and nominal target/functional annotation shown on left along with chemical structure and physico-chemical properties. The 8-point dose response curves for each hit with estimated Hill slope, EC50 and Efficacy [% max induction] values are shown on the right. Two data points per concentration (n = 2); data points are Mean ± SD. A 4-parameter logistic fit has been performed using R package: dr4pl. CC50 values for HEK293 and HepG2 CellTiter-Glo cytotoxicity assays performed by CALIBR (www.reframedb.org) are shown in the right hand section. Note: 0 = inactive in cytotoxicity assay. Physicochemical properties were calculated using RDKit, Python and KNIME: SlogP = partition coefficient (Wildman and Crippen, 1999); TPSA is the Topological Polar Surface Area (Ertl et al., 2000); MW is the exact Molecular weight; QED = Quantitative Estimate of Drug-likeness (Bickerton et al., 2012). See Figure 3—source data 3 along with Figure 3—source code 2 . [file elife-51739-fig3-data2.pdf]

Figure 3-figure supplement 1

Tyrothricin
Antibiotic

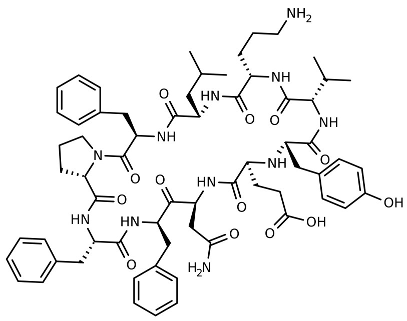

MW (RDKit)
1227.633

QED (RDKit)
0.063

SlogP (RDKit)
1.138

TPSA (RDKit)
379.75

RFM-011-941-5
Hill slope: 3.133 *EC50: 1.675 µM*

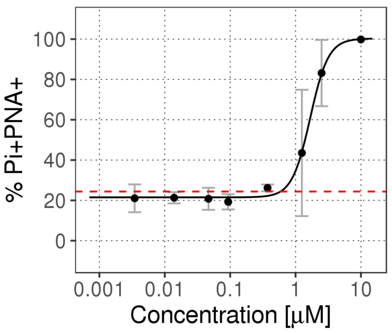

EC50 [µM]
1.67

Efficacy [% induction]
84

Hek CC50 [µM]
1.791

HepG2 CC50 [µM]
0

Mln 576
DNA topoisomerase II inhibitor

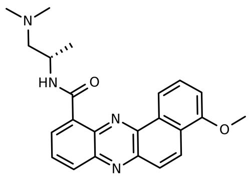

MW (RDKit)
388.19

QED (RDKit)
0.418

SlogP (RDKit)
3.625

TPSA (RDKit)
67.35

RFM-008-110-7
Hill slope: 0.846 *EC50: >10 µM*

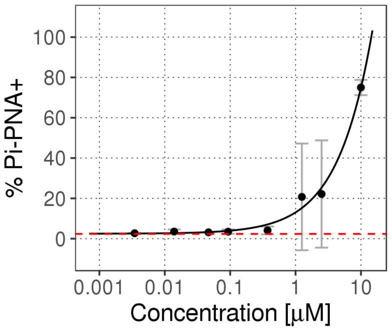

EC50 [µM]
>10

Efficacy [% induction]
76

Hek CC50 [µM]
0.686

HepG2 CC50 [µM]
0.574

Th-9402
Immunosuppressant

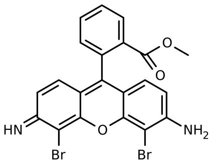

MW (RDKit)
499.937

QED (RDKit)
0.213

SlogP (RDKit)
5.578

TPSA (RDKit)
89.31

RFM-006-521-4
Hill slope: 4.118 *EC50: 0.404 µM*

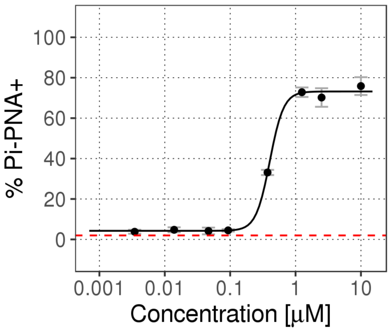

EC50 [µM]
0.4

Efficacy [% induction]
76

Hek CC50 [µM]
0

HepG2 CC50 [µM]
0

Topixantrone
DNA topoisomerase II inhibitor

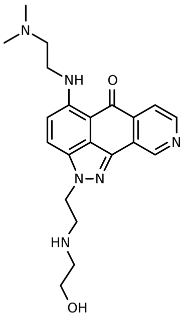

MW (RDKit)
394.212

QED (RDKit)
0.367

SlogP (RDKit)
1.198

TPSA (RDKit)
95.31

RFM-009-126-9
Hill slope: 3.952 *EC50: 0.491 µM*

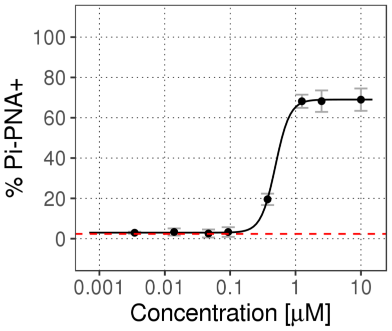

EC50 [µM]
0.49

Efficacy [% induction]
72

Hek CC50 [µM]
1.443

HepG2 CC50 [µM]
0.658

Imidazoacridinone
DNA topoisomerase II inhibitor

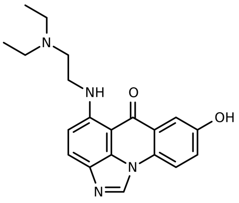

MW (RDKit)
350.174

QED (RDKit)
0.523

SlogP (RDKit)
2.898

TPSA (RDKit)
69.87

RFM-003-263-3
Hill slope: 3.469 *EC50: 3.853 µM*

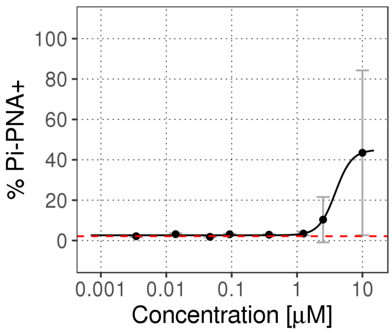

EC50 [µM]
3.85

Efficacy [% induction]
71

Hek CC50 [µM]
0.817

HepG2 CC50 [µM]
0.476

Proflavine
DNA synthesis interchalator

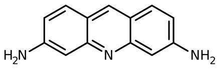

MW (RDKit)
209.095

QED (RDKit)
0.441

SlogP (RDKit)
2.552

TPSA (RDKit)
64.93

RFM-001-043-5
Hill slope: 2.271 *EC50: 2.739 µM*

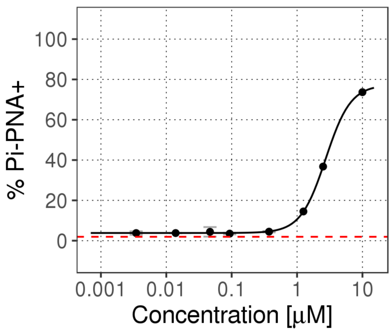

EC50 [µM]
2.74

Efficacy [% induction]
71

Hek CC50 [µM]
0

HepG2 CC50 [µM]
0

Manoalide
Ornithin Decarboxylase/PLA2 inhibitor

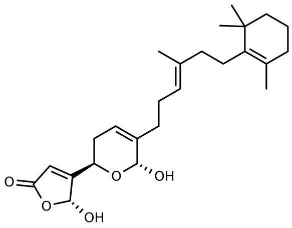

MW (RDKit)
416.256

QED (RDKit)
0.458

SlogP (RDKit)
4.855

TPSA (RDKit)
75.99

RFM-006-618-2
Hill slope: 2.530 *EC50: 4.682 µM*

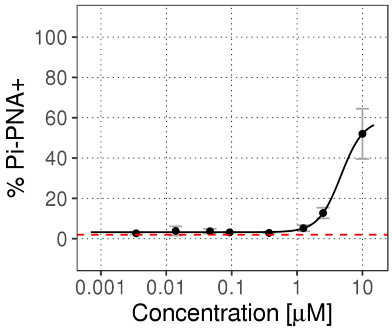

EC50 [µM]
4.68

Efficacy [% induction]
59

Hek CC50 [µM]
2.781

HepG2 CC50 [µM]
3.522

Merbromin
Carbonic anhydrase II inhibitor

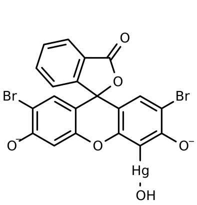

MW (RDKit)
703.841

QED (RDKit)
0.309

SlogP (RDKit)
2.542

TPSA (RDKit)
101.88

RFM-000-317-8
Hill slope: 1.972 *EC50: 2.866 µM*

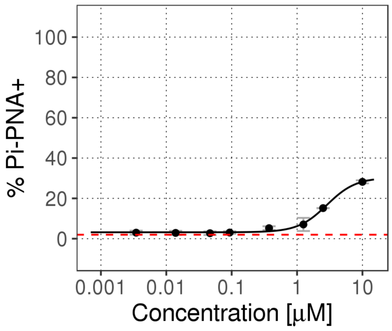

EC50 [µM]
2.86

Efficacy [% induction]
27

Hek CC50 [µM]
0

HepG2 CC50 [µM]
0

Acranil
Antiprotozoal

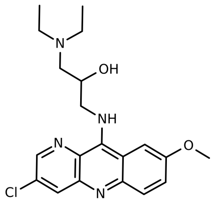

MW (RDKit)
387.171

QED (RDKit)
0.568

SlogP (RDKit)
4.165

TPSA (RDKit)
57.62

RFM-000-968-7
Hill slope: 3.134 *EC50: >10 µM*

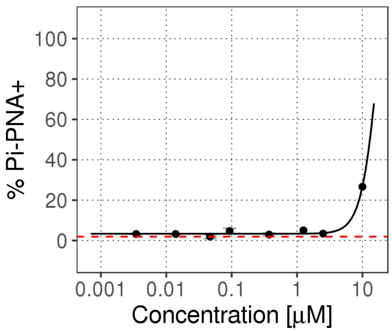

EC50 [µM]
>10

Efficacy [% induction]
25

Hek CC50 [µM]
1.136

HepG2 CC50 [µM]
2.827
