# Supplementary material for: A phenotypic screening platform utilising human spermatozoa identifies compounds with contraceptive activity
Source: eLife. 2020 Jan 28;9:e51739. doi: 10.7554/eLife.51739 (PMC7046468; doi:10.7554/eLife.51739)
Supplement: Supplementary file 1. — Summary of dose response experiments of primary motility hits with estimated EC50 and Efficacy [% reduction] values. Information and names were provided by Calibr. See Source data 1. [file elife-51739-supp1.docx]

**Supplementary File 1**

| *Name* | *EC50*  *[µM]* | *Efficacy [% reduction]* | *Information* |
| --- | --- | --- | --- |
| Kf-4939 | 0.49 | 100 | Platelet aggregation inhibitor |
| Mercufenol Chloride | 1.45 | 98 | Antiseptic |
| Bismuth Ethanedithiol | 2.5 | 98 | NA |
| Tyrothricin | 0.34 | 97 | Antibiotic |
| Ticlatone | 4.43 | 96 | Antibiotic |
| Phenylmercuric Borate | 1.59 | 91 | Antiseptic |
| Alexidine | > 10 | 86 | Phospholipase inhibitor |
| Disulfiram | > 10 | 70 | Aldehyde dehydrogenase 2 inhibitor |
| Octenidine | 6.85 | 54 | Antibacterial |
| Pfk-015 | > 10 | 47 | PFKFB3 inhibitor |
| Methylprednisolone Aceponate | 2.52 | 45 | Antiinflammatory |
| Zoticasone Propionate | 2.38 | 39 | Glucocorticoid receptor agonist |
| Brigatinib*^#^*  Fluorometholone Acetate  Amibegron Hydrochloride  Kf-41399  Uprosertib  Halobetasol Propionate  Pirtenidine  Lx-7101  Ap26113-analog  Methylbenzethonium Chloride  Cabazitaxel  Resiquimod  Oxyphenbutazone  Skq1  Docetaxel  Lestaurtinib  Brigatinib*^#^* | 0.68 3.3  0.22  4.43 > 10  > 10  2.66  > 10  1.35 > 10  > 10  3.22  4.08  > 10  0.05  4.97  1.89 | 38  35  31  31  31  30  28  28  24  24  23  22 21  21  20  17  15 | ALK receptor tyrosine kinase inhibitor  Antiinflammatory beta3-Adrenoceptor Agonist Chemoprotective agent  PKB alpha/Akt1 inhibitor  Antisporiatic  Antibiotic  LIM Domain Kinase inhibitor  ALK receptor tyrosine kinase inhibitor  Antiseptic  Tubulin inhibitor  Immunostimulant  Treatment of Gout  Neurologic drug  Tubulin inhibitor  Flt3 inhibitor  ALK receptor tyrosine kinase inhibitor |

*# from two different vendors; Information provided by Calibr; NA ... No Annotation*
