# Supplementary material for: A phenotypic screening platform utilising human spermatozoa identifies compounds with contraceptive activity
Source: eLife. 2020 Jan 28;9:e51739. doi: 10.7554/eLife.51739 (PMC7046468; doi:10.7554/eLife.51739)
Supplement: Supplementary file 2. — Summary of dose response experiments of primary acrosome hits with estimated EC50 and Efficacy [% increase] values. Information and names were provided by Calibr. Note that none of these compounds passed orthogonal counter screening and are considered as assay interfering compounds/false positives. See Source data 2. [file elife-51739-supp2.docx]

**Supplementary File 2**

| *Name* | *EC50*  *[µM]* | *Efficacy [% induction]* | *Information* |
| --- | --- | --- | --- |
| Tyrothricin | 1.67 | 84 | Antibiotic |
| Th-9402 | 0.4 | 76 | Immunosuppressant |
| Mln 576 | >10 | 76 | DNA topoisomerase II inhibitor |
| Topixantrone | 0.49 | 72 | DNA topoisomerase II inhibitor |
| Proflavine | 2.74 | 71 | DNA synthesis interchalator |
| Imidazoacridinone | 3.85 | 71 | DNA topoisomerase II inhibitor |
| Manoalide | 4.68 | 59 | Ornithin Decarboxylase/PLA2 inhibitor |
| Merbromin | 2.86 | 27 | Carbonic anhydrase II inhibitor |
| Acranil | 21.61 | 25 | Antiprotozoal |

*Information provided by Calibr*
